# Supplementary material for: Quinolone and Organophosphorus Insecticide Residues in Bivalves and Their Associated Risks in Taiwan
Source: Molecules. 2020 Aug 10;25(16):3636. doi: 10.3390/molecules25163636 (PMC7465073; doi:10.3390/molecules25163636)
Supplement: Supplementary file 1 [file molecules-25-03636-s001.pdf]

molecules

Article

Quinolone and Organophosphorus Insecticide Residues in Bivalves and Their Associated Risks in Taiwan

Ching-Feng Wu [1,†], Ching-Hung Chen [2,†], Ching-Yang Wu [1], Chen-Si Lin [3], Yao-Chi Su [4], Ching-Fen Wu [4], Hsiao-Pei Tsai [5,6], Pei-Shan Fan [4], Chang-Hui Yeh [4], Wei-Cheng Yang [3,*] and Geng-Ruei Chang [4,5,6,*]

[1] Division of Thoracic and Cardiovascular Surgery, Department of Surgery, Chang Gung University, Chang Gung Memorial Hospital, Linkou, 5 Fuxing Street., Guishan District, Taoyuan 33305, Taiwan; maple.bt88@gmail.com (C.F.W.); wu.chingyang@gmail.com (C.Y.W.)
[2] Department of Anesthesiology, Show Chwan Memorial Hospital, 1 Section, 542 Chung-Shan Road, Changhua 50008, Taiwan; consideration00@gmail.com (C.H.C.)
[3] Department of Veterinary Medicine, School of Veterinary Medicine, National Taiwan University, 4 Section. 1 Roosevelt Road, Taipei 10617, Taiwan; cslin100@ntu.edu.tw (C.S.L.); yangweicheng@ntu.edu.tw (W.C.Y.)
[4] Department of Veterinary Medicine, National Chiayi University, 580 Xinmin Road, Chiayi 60054, Taiwan; shu@mail.ncyu.edu.tw (Y.C.S.); cfwu@mail.ncyu.edu.tw (C.F.W.); babybelle349@gmail.com (P.S.F.); grchang@mail.ncyu.edu.tw (G.R.C.)
[5] Veterinary Teaching Hospital, National Chiayi University, 580 Xinmin Road, Chiayi 60054, Taiwan; tsaibelle@mail.ncyu.edu.tw (H.P.T.) ; (G.R.C.)
[6] Ph.D. Program of Agriculture Science, National Chiayi University, 300 Syuefu Road, Chiayi 60004 and Taiwan
* Correspondence: yangweicheng@ntu.edu.tw (W.C.Y.), grchang@mail.ncyu.edu.tw (G.R.C.); Tel.: +886-2-33663871 (W.C.Y.), +886-5-2732946 (G.R.C.)

† These authors contributed equally to this work.

Supplementary Materials

Table S1. Recovery, repeatability, and limit of quantification of quinolones spiked into bivalve samples

Analyte	Spikedlevel (ng/g)	Hard clam Recovery (%)	Hard clam RSD (%)	Freshwater clam Recovery (%)	Freshwater clam RSD (%)	LOQ (ng/g)
Ciprofloxacin	5	98.52	5.27	97.36	3.71	5
	25	99.89	6.72	98.59	5.32	
Danofloxacin	5	98.15	6.17	99.36	5.42	5
	25	99.47	7.96	99.58	8.35	
Difloxacin	5	96.38	8.32	97.21	7.41	5
	25	98.71	8.95	98.56	8.23	
Enrofloxacin	5	100.52	2.83	99.17	10.15	5
	25	102.57	3.58	100.46	9.81	
Fleroxacin	5	99.52	7.83	98.62	6.72	5

	25	100.65	6.56	99.86	8.29	
Flumequine	5	98.56	3.82	99.23	9.21	5
	25	99.16	5.61	100.72	8.53	
Marbofloxacin	5	96.35	8.57	95.47	9.36	5
	25	97.68	9.13	98.72	11.35	
Sarafloxacin	5	99.62	5.73	98.48	7.73	5
	25	100.76	9.25	99.39	8.39	

Table S2. Recovery, repeatability, and limit of quantification of organophosphorus insecticides spiked into bivalve samples

Analyte	Spikedlevel (ng/g)	Hard clam		Freshwater clam		LOQ (ng/g)
		Recovery (%)	RSD (%)	Recovery (%)	RSD (%)	
Fenamiphos	5	98.52	5.27	97.36	3.71	5
	25	99.89	6.72	98.59	5.32	
Fenthion	5	98.15	6.17	99.36	5.42	5
	25	99.47	7.96	99.58	8.35	
Methamidophos	5	96.38	8.32	97.21	7.41	5
	25	98.71	8.95	98.56	8.23	
Phoxim	5	100.52	2.83	99.17	10.15	5
	25	102.57	3.58	100.46	9.81	
Profenophos	5	99.52	7.83	98.62	6.72	5
	25	100.65	6.56	99.86	8.29	
Trichlorfon	5	98.56	3.82	99.23	9.21	5
	25	99.16	5.61	100.72	8.53	
Chlorpyrifos	5	96.35	8.57	95.47	9.36	5
	25	97.68	9.13	98.72	11.35	
Diazinon	5	99.62	5.73	98.48	7.73	5
	25	100.76	9.25	99.39	8.39	
Fenitrothion	5	103.31	7.68	105.06	6.53	5
	25	107.26	5.37	108.37	8.16	
Formothion	5	105.72	6.51	102.61	5.32	5
	25	108.61	9.82	104.37	7.85	
Malathion	5	107.42	10.31	105.82	8.67	5
	25	113.56	12.67	108.35	10.23	
Triazophos	5	112.32	8.27	108.32	7.35	5
	25	114.53	7.36	110.48	9.18	

Table S3. MS/MS fragmentation conditions for LC-amenable 8 quinolones

Analyte	Retention Time	Quantification ion (Collision energy) m/z (eV)	Identification ion (Collision energy) m/z (eV)
Ciprofloxacin	9.66±0.02	332→314 (8)	332→231 (11)
Danofloxacin	10.37±0.02	358→340 (9)	358→283 (18)
Difloxacin	11.13±0.02	400→356 (10)	400→299 (17)
Enrofloxacin	10.21±0.02	360→316 (6)	360→245 (8)
Fleroxacin	7.98±0.02	370→326 (25)	370→269 (19)
Flumequine	15.93±0.02	262→244 (14)	262→202 (14)
Marbofloxacin	7.79±0.02	363→345 (17)	363→72 (26)
Sarafloxacin	11.78±0.02	386→368 (12)	386→342 (16)

Table S4. MS/MS fragmentation conditions for LC- or GC-amenable 12 organophosphorus insecticides

Analyte	LC/GC	Retention Time	Quantification ion (Collision energy) m/z (eV)	Identification ion (Collision energy) m/z (eV)
Fenamiphos	LC	10.31±0.02	304→202 (37)	304→217 (24)
Fenthion	LC	10.84±0.02	279→169 (20)	279→247 (14)
Methamidophos	LC	2.74±0.02	142→94 (14)	142→125 (13)
Phoxim	LC	10.99±0.02	299→129 (10)	299→153 (10)
Profenophos	LC	11.45±0.02	373→128 (55)	373→303 (19)
Trichlorfon	LC	4.34±0.02	257→79(28)	257→109 (18)
Chlorpyrifos	GC	10.45±0.02	314→258 (15)	314→286 (5)
Diazinon	GC	8.75±0.02	304→179 (15)	304→162 (5)
Fenitrothion	GC	10.16±0.02	277→109 (20)	277→260 (5)
Formothion	GC	9.33±0.02	224→125 (20)	224→155 (10)
Malathion	GC	10.26±0.02	173→127 (5)	173→99 (15)
Triazophos	GC	13.26±0.02	257→162 (5)	257→119 (30)

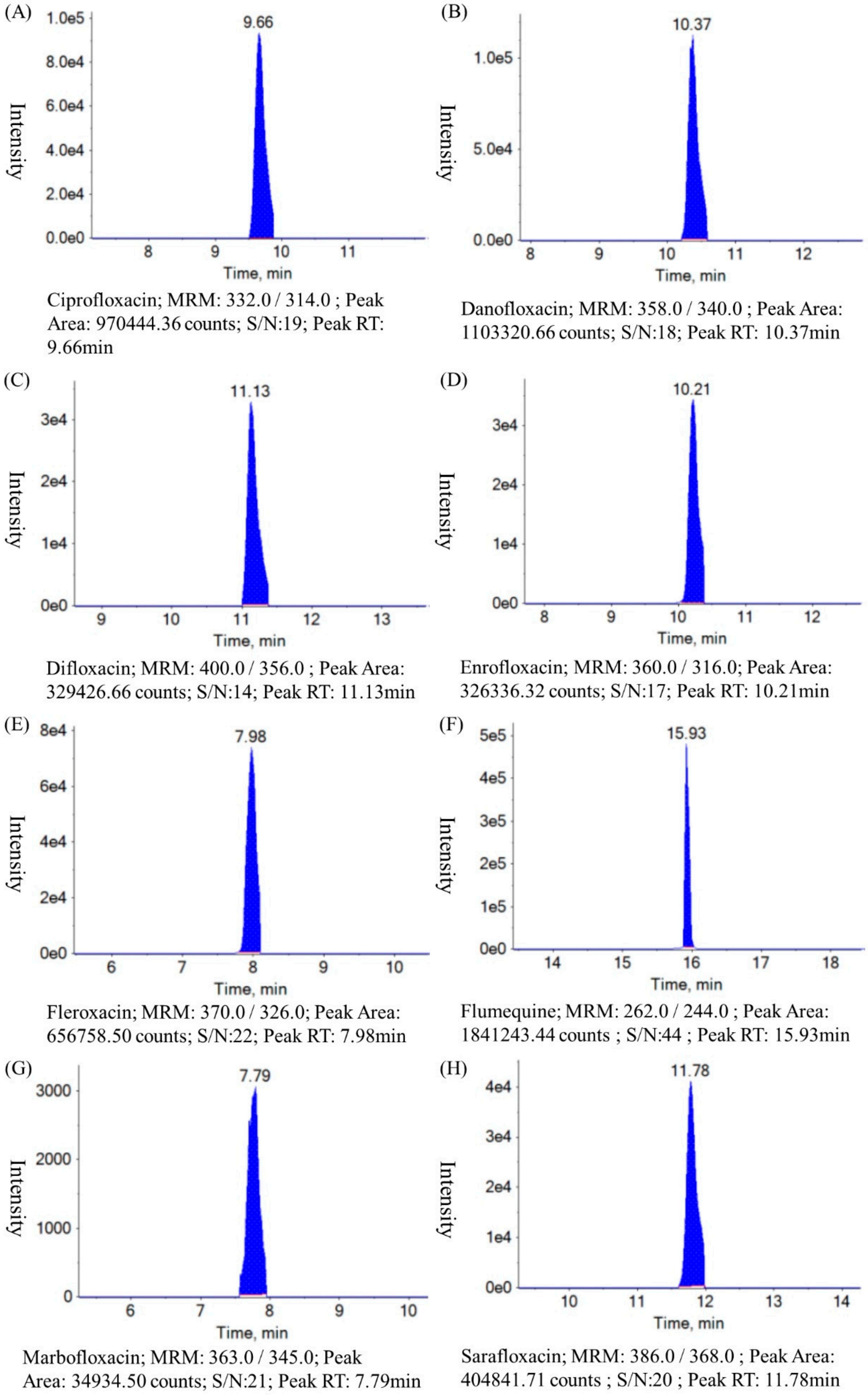

(A) Ciprofloxacin; MRM: 332.0 / 314.0 ; Peak Area: 970444.36 counts; S/N:19; Peak RT: 9.66min

(B) Danofloxacin; MRM: 358.0 / 340.0 ; Peak Area: 1103320.66 counts; S/N:18; Peak RT: 10.37min

(C) Difloxacin; MRM: 400.0 / 356.0 ; Peak Area: 329426.66 counts; S/N:14; Peak RT: 11.13min

(D) Enrofloxacin; MRM: 360.0 / 316.0; Peak Area: 326336.32 counts; S/N:17; Peak RT: 10.21min

(E) Fleroxacin; MRM: 370.0 / 326.0; Peak Area: 656758.50 counts; S/N:22; Peak RT: 7.98min

(F) Flumequine; MRM: 262.0 / 244.0 ; Peak Area: 1841243.44 counts ; S/N:44 ; Peak RT: 15.93min

(G) Marbofloxacin; MRM: 363.0 / 345.0; Peak Area: 34934.50 counts; S/N:21; Peak RT: 7.79min

(H) Sarafloxacin; MRM: 386.0 / 368.0 ; Peak Area: 404841.71 counts ; S/N:20 ; Peak RT: 11.78min

Figure S1. LC-MS/MS chromatogram of the detected quinolones at the quantification ion at LOQ (5 ng/g) in the bivalve samples.

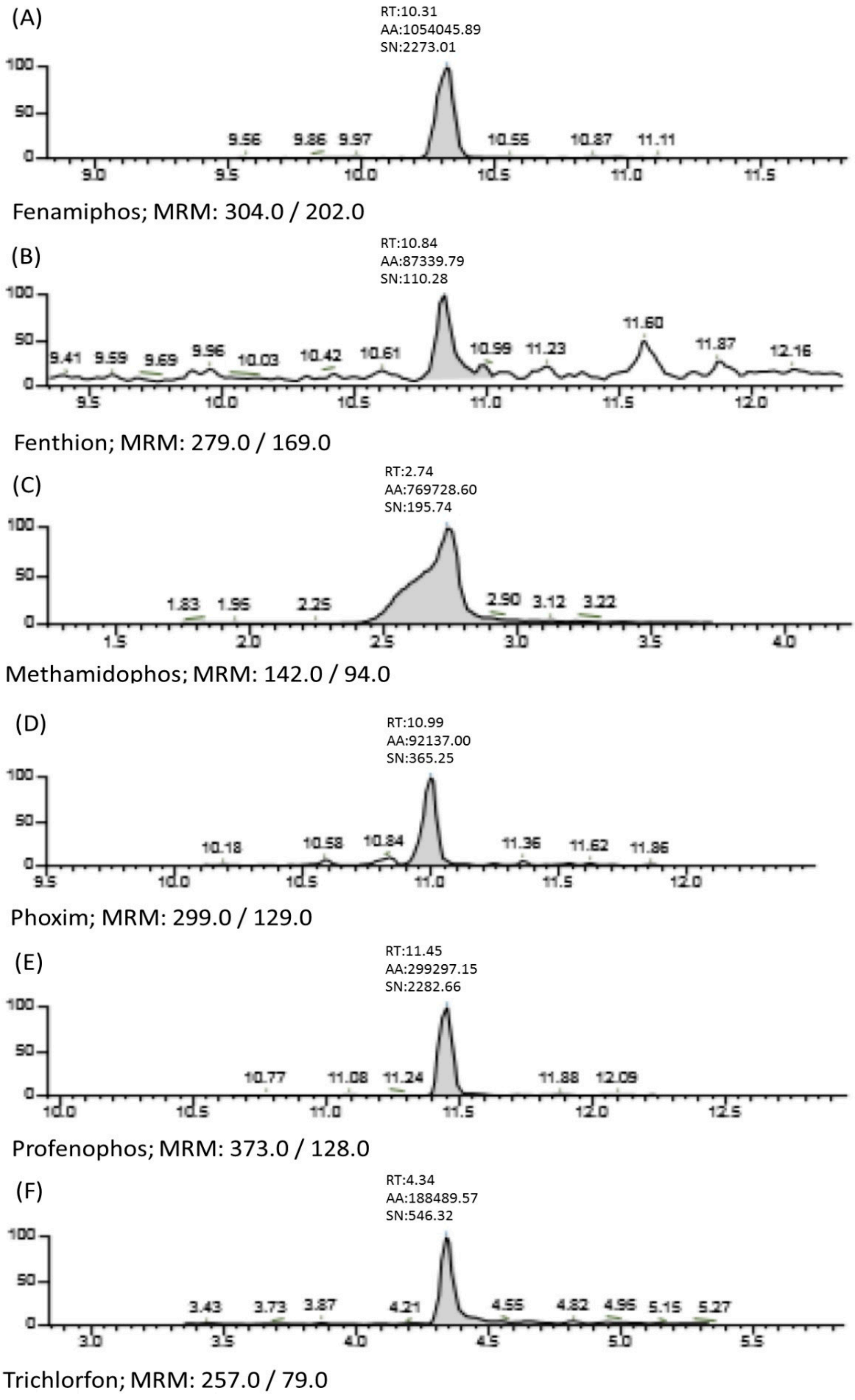

Figure S2. LC-MS/MS chromatograms of the detected organophosphorus insecticide at the quantification ion at LOQ (5 ng/g) in the bivalve samples.

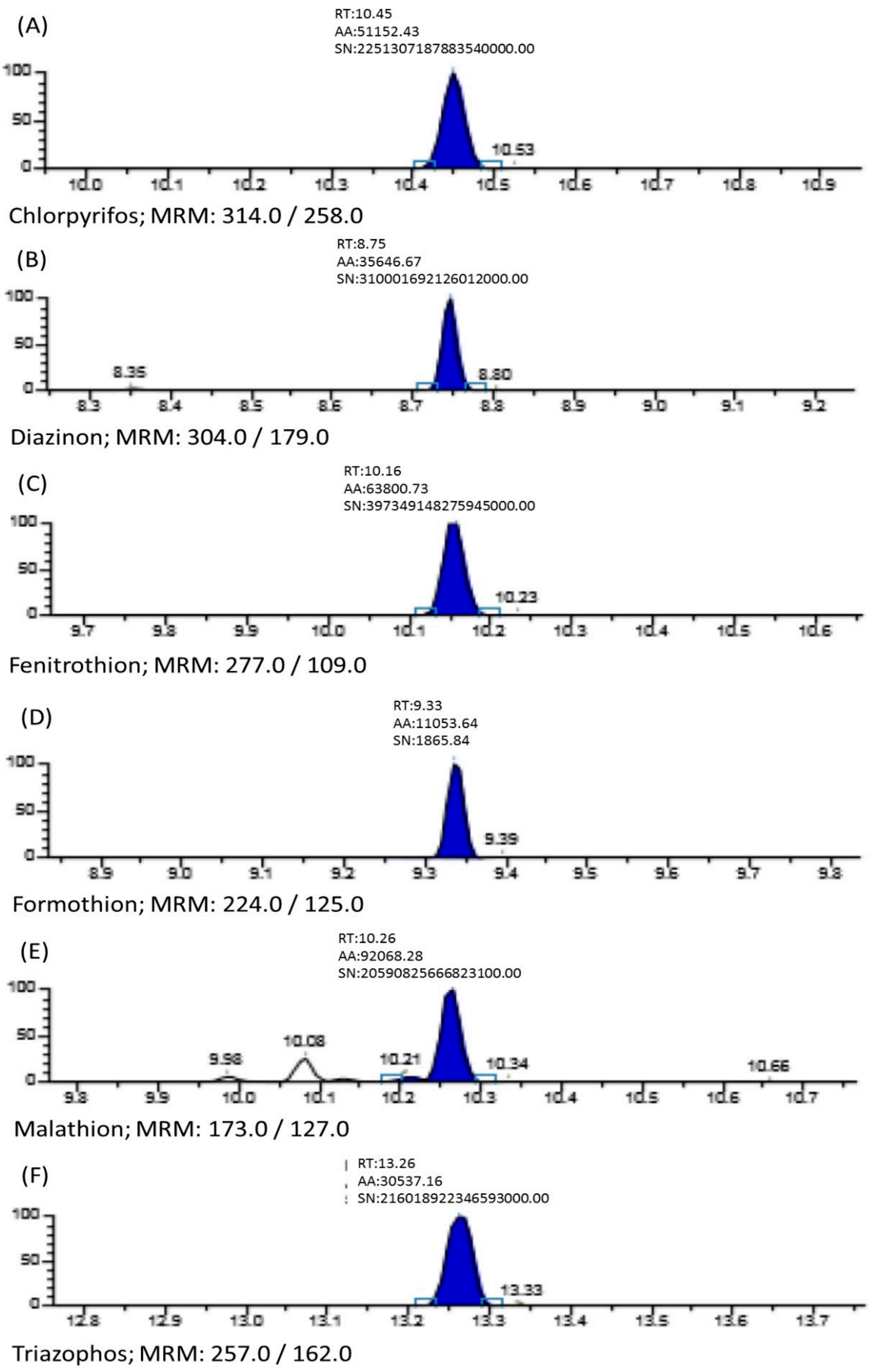

Figure S3. GC-MS/MS chromatograms of the detected organophosphorus insecticide at the quantification ion at LOQ (5 ng/g) in the bivalve samples.
